# Editorial: Novel approaches to prevent and treat intracellular bacterial infections

**DOI:** 10.3389/fmicb.2023.1249934

**Published:** 2023-07-24

**Authors:** Volker Behrends, Jesús Navas, Izabela Korona-Glowniak, Michal Letek

**Affiliations:** ^1^School of Life and Health Sciences, University of Roehampton London, London, United Kingdom; ^2^Departamento de Biología Molecular, University of Cantabria, Santander, Spain; ^3^Department of Pharmaceutical Microbiology, Medical University of Lublin, Lublin, Poland; ^4^Departamento de Biología Molecular, Universidad de León, León, Spain

**Keywords:** intracellular, bacteria, infection, treatment, antibiotics

Infectious diseases caused by intracellular bacterial pathogens present a considerable health threat, requiring innovative strategies to overcome the limitations of traditional antibiotics, which cannot easily penetrate and accumulate inside host cells. This Research Topic brings together a collection of papers that explore “*Novel approaches to prevent and treat intracellular bacterial infections*”, highlighting common themes that emerge from the research.

Two studies focus on the discovery and evaluation of alternative antimicrobial agents from natural products. Zhong et al. investigate the activity of essential oil from satsuma mandarin against *Aeromonas hydrophila*, showing that it disrupts the extracellular membrane permeability, while Sawicki et al. explore the impact of propolis treatment on the metabolic pathways and cell envelope of *Mycobacterium tuberculosis*.

Targeting the bacterial cell wall is another common theme. Zhou et al. provide an extensive review of various targets within the cell wall, highlighting their significance in bacterial growth and virulence. The authors emphasize the need for innovative methodologies to discover new antibiotics that target the cell wall components effectively.

Addressing the challenges of antibiotic resistance and treatment failures, Hou et al. review the use of non-antibiotic compounds for preventing and suppressing chlamydial infections. This approach aims to identify alternative strategies beyond traditional antibiotic treatments, considering the potential risks of antibiotic resistance.

Two studies explore the utilization of nanoparticles for intracellular infection treatment. Zhang et al. develop composite nanoparticles loaded with cellulase and levofloxacin, combined with ultrasound irradiation, to target *Mycobacterium tuberculosis* biofilms. Du et al. investigate the effectiveness of silver nanoparticles in treating multidrug-resistant *Escherichia coli* infections in mice. Both studies highlight the potential of nanotechnology-based approaches for improved bacterial infection management.

Overall, the findings presented in this Research Topic have the potential to shape the future of intracellular bacterial infection management and contribute to the global fight against antimicrobial resistance. We commend the authors for their valuable contributions and invite readers to delve into these exciting research findings.

## Author contributions

VB wrote the first draft of the manuscript. All authors contributed to the manuscript revision and have read and agreed to the published version of the manuscript.

